# Subclinical Ocular Motility Dysfunction and Extraocular Muscle Changes in Inactive Graves’ Orbitopathy

**DOI:** 10.3390/jpm14080848

**Published:** 2024-08-10

**Authors:** Filippo Lixi, Alberto Cuccu, Giuseppe Giannaccare, Matteo Onnis, Mihaela Madalina Timofte Zorila, Stefano Mariotti, Rosanna Vacca, Paola Elisa Meloni, Michela Pisu, Chiara Mura, Francesco Boi

**Affiliations:** 1 Eye Clinic, Department of Surgical Sciences, University of Cagliari, 09124 Cagliari, Italy; f.lixi1@studenti.unica.it (F.L.); a.cuccu@aoucagliari.it (A.C.); m.onnis15@studenti.unica.it (M.O.); 2 Department of Ophthalmology, Cai Ferate Clinical Hospital, 1 Garabet Ibraileanu Street, 700506 Iasi, Romania; mihaela-madalina.timofte-zorila@d.umfiasi.ro; 3 Faculty of Medicine, “Grigore T. Popa” University of Medicine and Pharmacy, 16 Universitatii Street, 700115 Iasi, Romania; 4 Endocrinology Unit, Department of Medical Sciences and Public Health, University of Cagliari, 09124 Cagliari, Italy; mariotti@medicina.unica.it (S.M.); rosanna.vacca23@gmail.com (R.V.); dottoressapisu@gmail.com (M.P.); chiara.mura@unica.it (C.M.); francesco.boi@unica.it (F.B.)

**Keywords:** thyroid eye disease, Graves’ orbitopathy, Red Filter Test, CAS

## Abstract

This study aimed to investigate the presence of structural and functional changes in extraocular muscles (EMs) among patients with inactive Graves’ orbitopathy (GO) classified according to the Clinical Activity Score (CAS). Sixty-seven patients with Graves’ disease (GD) and inactive GO were included. The data collected included clinical parameters, thyroid function, autoantibody levels, EOM morphology via orbital ultrasound (US), and ocular motility. Patients were stratified into Red Filter Test (RFT)-positive or RFT-negative groups based on the presence or absence of latent diplopia during the RFT examination. Thirty-three patients (49.25%) exhibited latent diplopia on the RFT, despite not reporting double vision during standard ocular motility tests. Significant differences were observed between the two groups in terms of age, disease duration, intraocular pressure (IOP) elevation in up-gaze, and medial rectus muscle thickness (*p* < 0.05). No significant differences were found in thyroid status, TRAb and ATA levels, CASs, exophthalmos, or lateral rectus thickness between the two groups. This study revealed that in inactive GO, subclinical EM dysfunction and morphological changes may be present, which might not be apparent through routine ocular examinations. The RFT is effective in detecting latent diplopia, highlighting its utility in identifying subtle ocular motility issues and subclinical muscle involvement. Comprehensive evaluations combining functional tests like the RFT and imaging are essential for early detection of GO-related abnormalities, enabling tailored and prompt management and improving patient outcomes.

## 1. Introduction

Graves’ Disease (GD) is an autoimmune disorder, known as the leading cause of hyperthyroidism, responsible for 50–80% of cases globally [1,2,3]. The disease is typically triggered by thyrotropin receptor autoantibodies (TRAb), which bind to and activate the thyroid-stimulating hormone receptor (TSHR), causing an overproduction of thyroid hormones [4].

Graves’ orbitopathy (GO) is the most frequent extra-thyroidal manifestation of GD, affecting about 50% of those with Graves’ hyperthyroidism. It is noteworthy that individuals with Hashimoto’s thyroiditis or even those without evident thyroid disease can also develop GO [5,6].

As an immune-mediated disease, GO is triggered by auto-reactive T lymphocytes recognizing one or more antigens shared by the thyroid and orbital tissues, primarily the TSHR [7]. This inflammatory process causes the expansion of orbital fibro-adipose tissue, the infiltration and enlargement of extraocular muscles (EMs), leading to clinical manifestations such as exophthalmos, diplopia, ocular soft tissue changes, and possible optic nerve compression [8,9].

The Clinical Activity Score (CAS), proposed by Mourits and colleagues, is based on a set of criteria, with each criterion being scored as present or absent, and aims to distinguish patients with active GO from those with inactive disease [10].

However, it is well known that GD patients with inactive GO may present morphologic alterations of EMs and/or fibro-adipose tissue changes detectable with different imagine techniques [11]. Moreover, the dysfunction of EMs can cause eye movement-related impairment, significantly altering the quality of patients’ life and presenting as a treatment challenge [12]. 

This study aimed to investigate the presence of alteration in EMs’ function, evaluated by means of the Red Filter test (RFT) among patients with inactive Graves’ orbitopathy (GO) without frank diplopia. 

## 2. Materials and Methods

This was a retrospective study conducted at the University Hospital of Cagliari, Italy. The study received approval by the Institutional Review Board (IRB) of the University of Cagliari (PG/2015/3455; 26 February 2015). All subjects provided written informed consent and the study was performed in accordance with the Declaration of Helsinki.

### 2.1. Patients’ Selection and Data Collection

A total of 67 consecutive patients with GD and inactive GO according to CAS were examined. Patients were enrolled consecutively between January 2011 and December 2014 at a single center (University Hospital of Cagliari, Cagliari, Italy). The following data were collected from each patient’s medical file: age, sex, disease duration, CAS class, free thyroxine (FT4), free triiodothy-ronine (FT3) and thyroid-stimulating hormone (TSH), thyrotropin receptor antibody (TRAb) [TRAb levels were considered as a low titer (1–2 IU/L), moderate titer (2–10 IU/L) and high titer (>10 IU/L)], Anti-Thyroid Antibody (ATA) levels [ATA levels were considered positive if anti-thyroglobulin (TG) antibodies were equal to or higher than 4.5 IU/mL, or if anti-thyroid peroxidase (TPO) antibodies were equal to or higher than 60 IU/mL], intraocular pressure (IOP) measurement in primary and up-gaze for both eyes, best-corrected visual acuity (BCVA), slit lamp examination, eye motility, RFT and exophthalmometry. Patients were categorized into 2 groups based on positive (RFT-positive) or negative diplopia (RFT-negative) on RFT.

### 2.2. Ophthalmic Assessment

Ocular motility tests were performed by the same experienced ophthalmologist (A.C.) to evaluate latent or manifest deficits. Manifest diplopia was assessed by asking the patients to follow a small tool moved from the center of the visual field towards its periphery in the four main directions. The presence of latent diplopia was investigated using the RFT: a red glass was placed in front of the patient’s right eye, and the patient was asked to focus on a single white light source directly in front of them, maintaining a primary gaze. The patient was then instructed to look at the light source from various positions of gaze, including the primary position and the eight cardinal positions. If there was a muscle dysfunction, the patient reported diplopia (perception of two lights: white and red). The direction of gaze that resulted in diplopia or showed the greatest separation of the images could suggest which structures were involved. IOP was measured first in primary gaze and then in up-gaze. The measurement of eye proptosis was assessed with Hertel’s exophtalmometer. The normal range of values was considered 12–21 mm. Values above the upper limit or the difference between the two eyes ≥ 3 mm were considered positive.

### 2.3. Imaging Evaluation

Orbital US was performed by the same experienced echographer (A.C.) using a B-mode MYLAB 70XV Echo-scan (ESAOTE, Genova, Italy) with a 10 MHz transducer. The medial and lateral rectus muscles were examined in both eyes, while the vertical recti were measured only occasionally. During the examination, the patients were asked to look straight ahead, maintaining a primary gaze position. The probe was placed on the closed eyelid, on the opposite side of the muscle that was examined. Suitable sections were frozen on the screen when the trans-bulbar muscle stripe appeared as distinctly as possible. The thickest section of the muscle was then measured at the point of greatest enlargement, perpendicular to the muscle axis, using calipers. Muscles were considered normal if the thickness was < 4 mm, thickened if the thickness was between 4 and 6 mm, and very thickened if the thickness was > 6 mm.

### 2.4. Statistical Analysis

Statistical analysis was performed using the Statistical Package for Social Science software (IMB SPSS Statistics, version 25 for Windows). Means ± standard deviations (SDs)/median for continuous variables were estimated or percent distributions were presented. The distribution of variables was assessed with Shapiro–Wilk tests. Visual acuity was converted in logMAR for statistical analysis. Fisher’s test and Chi-squared test were used to compare categorical variables. Parametric (*t*-test) and non-parametric (Mann–Whitney U test) tests were used to compare normally and non-normally distributed variables, respectively, between groups. A *p* value of <0.05 was considered statistically significant.

## 3. Results

### 3.1. Demographic, Laboratories and Clinical Characteristics of Study Population

All patients were Caucasians. A total of 134 eyes of 67 patients were included in this study. The medical records of 46 females and 21 males with a mean age 46.0 ± 12.1 (range 18–72 years) were evaluated. Thirty-six (53.73%) patients were hyperthyroid, twenty-eight (41.79%) were euthyroid, and three (4.47%) were hypothyroid. The mean duration of thyroid disease was 29.00 ± 41.21 months. TSH mean value was 1.57 mU/L (median 0.50 mU/L), FT4 mean value was 2.45 ng/dL (median 1.40 ng/dL), and FT3 mean value was 4.47 pg/mL (median 3.36 pg/mL). TRAb values were positive in 51 (76.11%) patients, with a low titer in 22 subjects (32.83%), moderate titer in 16 (23.88%) and high titer in 13 (19.40%). Sixteen (23.88%) patients were negative for TRAb. ATAs were positive in 48 (71.64%) and negative in 19 (28.36%) patients. All patients presented CAS ≤ 2. Thirty-six (53.73%) patients were CAS 0, twenty-six (38.80%) were CAS 1 and five (7.46%) were CAS 2. Eight (11.94%) patients presented mild exophthalmos (1–2 mm proptosis above the upper limit or 3–4 mm of difference compared to the fellow eye). No patients complained of diplopia upon evaluation of ocular motility. BCVA was logMAR 0.02 ± 0.09. Mean IOP in primary gaze was 14.46 ± 2.76 mmHg. Mean IOP in up-gaze was 16.64 ± 4.07. In the US exam, considering both orbits, medial rectus resulted normal in 69 (51.49%), thickened in 57 (42.54%) and very thickened in 8 eyes (5.97%). Lateral rectus was normal in 109 (81.34%), thickened in 22 (16.42%) and very thickened in 3 eyes (2.24%). During the ocular motility examination, no patients reported manifest diplopia. Conversely, in the RFT, 33 (49.25%) patients exhibited latent diplopia in the vertical gaze position.

Demographic, laboratory and clinical characteristics are displayed in Table 1. 

### 3.2. Analysis of RFT-Positive vs. RFT-Negative Patients

Study subjects were divided into two groups according to positive (RFT-positive, *n* = 33) or negative diplopia on RFT (RFT-negative, *n* = 34). The characteristics of two groups are displayed in Table 2.

The gender distribution was similar across both groups (*p* = 1). The RFT-positive group had a significantly higher mean age (49.1 ± 12.3 years vs. 43.0 ± 11.3 years, *p* = 0.03) and a longer mean disease duration (34.1 ± 37.6 months vs. 24.1 ± 44.5 months, *p* = 0.04). Thyroid status, thyroid hormone levels (TSH, FT4, FT3), TRAb levels, ATA, CASs, exophthalmos presence and BCVA showed no significant differences between the groups. The mean IOP exhibited a significant increase from 14.20 ± 2.80 mmHg in primary gaze to 18.15 ± 4.56 mmHg in up-gaze (*p* < 0.001, Mann–Whitney U test), corresponding to a 27.8% elevation in the RFT-positive group. Concerning the RFT-negative group, the mean IOP exhibited an increase from 14.71 ± 2.72 mmHg in primary gaze to 15.18 ± 2.87 mmHg in up-gaze (*p* = 0.35, Mann–Whitney U test), corresponding to a 3.2% elevation in the RFT-negative group. In up-gaze, the mean IOP was significantly higher in the RFT-positive group compared to the RFT-negative group (*p* = 0.00005). Ultrasound measurements revealed that the medial rectus muscle was more frequently thickened in the RFT-positive group (59.1% vs. 38.2%, *p* = 0.003), while the lateral rectus muscle thickness did not differ significantly between the groups (24.2% vs. 13.2, *p* = 0.10).

## 4. Discussion

External eye muscles play a critical role in the pathophysiology of GO, being involved in both active and inactive phases of the disease. In these conditions, the muscles may become edematous and infiltrated or undergo fibrosis, which can alter eye motility [12,13].

Double vision or diplopia is a common and debilitating symptom of GO [14]. The European Group on Grave’s Orbitopathy (EU-GOGO) reported that 49% of patients with GO presented some degree of eye movement dysfunction [15]. Additionally, patients with diplopia pose a major burden for public health systems due to potential occupational impairments, especially when double vision occurs in the primary and reading position [16].

Diplopia may be latent or manifest. The former occurs when the eyes tend to deviate from their normal position, but this misalignment is typically kept in check by the brain’s fusion mechanism. In latent diplopia, double vision does not usually occur because the brain compensates and corrects the misalignment. However, under certain conditions, the brain’s ability to maintain proper alignment can weaken, potentially leading to eye strain or double vision. Conversely, manifest diplopia is a condition where there is a constant and noticeable misalignment of the eyes, leading to persistent double vision which the brain cannot correct [17]. 

Several classifications have been developed and used to evaluate GO. VISA and EUGOGO both subjectively assess the presence of diplopia, noting whether it is vertical or horizontal, constant or intermittent, and if it worsens in the morning [18]. None of these scores can detect latent diplopia or measure the level of involvement of all EMs throughout the eye disease. This gap in the ocular evaluations highlights the need for some methods to be implemented in current practice to comprehensively assess diplopia. Accordingly, while the Hess Lancaster test is considered the gold standard for evaluating diplopia, it requires specialized equipment and trained technicians. In contrast, the RFT is a straightforward, cost-effective, and reliable method that can be easily performed in a clinical setting without specialized instruments [19]. Therefore, RFT can offer a valid alternative to rapidly and non-invasively evaluating diplopia in these patients.

In the studied populations, 33 (49.25%) patients with inactive GO exhibited positive diplopia on RFT in vertical gaze, which they had previously not complained of during the ocular motility examination. This could be due to the ability of RFT to evaluate the total deviation (both manifest and latent) by disrupting fusional mechanisms through stark color differences between the eyes [20]. This finding highlights that even in the absence of active disease, subclinical changes in EM function can still be present. Therefore, while CAS is an effective score for categorizing disease activity, it may not fully capture subtle changes in muscle morphology or function.

In terms of structural alterations, a significant difference in IOP between primary and up-gaze positions is well-established in patients with GO as an indicator of inferior rectus muscle thickening. Specifically, in the up-gaze position, the pulling of inferior rectus muscle in patients with GO causes the superior rectus muscle to use greater force to elevate the eyeball. This additional force acts on the eyeball wall and the compression of the superior and inferior rectus muscle significantly elevates the IOP [21]. Despite the lack of a thickness measurement of the vertical recti in our cohort due to different limitations of US in evaluating superior and inferior rectus [22], the significant IOP increment in the RF-positive group may reflect increased resistance from a stiffened inferior rectus muscle. 

Ultrasounds represent a cheap, rapid and effective tool for assessing eye muscle involvement in patients with GO, especially compared with more invasive exams like computed tomography (CT) and magnetic resonance imaging (MRI) [23].

B-scan images showed that the horizontal recti had different thickening rates between the two groups, with more thickening of the medial rectus in the RFT-positive group. This finding is consistent with the literature, which indicates that the medial rectus is primarily involved in GO, after the inferior rectus [24]. No significant difference was observed in the lateral rectus thickness between the two groups, likely because the latter is the least affected muscle in GO, followed by the oblique muscles. [25]. Despite these results, the RFT was positive in vertical gaze, likely because the maximum vertical disparity that can be fused is only about 3–5 Δ, significantly less than the horizontal fusion amplitude of 15–20 Δ [26].

The factors of disease duration and age of patients exhibited a significant difference between the two groups. These could lead to an increased cumulative exposure to autoimmune inflammation and subsequently more significant tissue damage and fibrosis over time, which could be reflected in imaging and functional test alterations. Additionally, age-related changes in the ocular muscles and overall tissue elasticity reduction could contribute to the observed differences between the groups [27].

Patients with GO may have hyperthyroidism, hypothyroidism, or euthyroidism [5]. The distribution of thyroid status did not significantly differ between the RFT-positive and RFT-negative groups, suggesting that latent diplopia is not directly influenced by the current thyroid status. Similarly, sex and patients with some degree of exophthalmos did also not differ between the two groups.

The role of TRAb and ATA in the pathogenesis of GD and GO has been extensively investigated [28]. However, the lack of a significant difference between the groups suggests that these antibodies alone do not determine the presence of diplopia, as already shown in previous reports [29,30].

These findings suggest that even in inactive GO, there can be subtle yet clinically relevant changes in EOM function and morphology. The early detection of these changes could significantly influence the progression and prognosis of the disease by facilitating a dedicated management plan to mitigate disease advancement. This plan might involve more rigorous follow-up schedules to monitor the patient closely and implement timely interventions. Preventive measures, such as protecting the eyes from sun and wind exposure and encouraging patients to quit smoking, can help reduce risk factors associated with disease exacerbation [31]. Ensuring optimal thyroid hormone levels by tailoring anti-thyroid therapy to achieve euthyroidism and avoiding hormonal fluctuations is also crucial in stabilizing the condition. Furthermore, considering selenium’s potential benefits in slowing disease progression, initiating therapy with 200 mcg/day could be a viable option for such patients [32]. Through these strategies, early intervention may improve disease progression and patient outcomes.

Nonetheless, the study had several limitations. Firstly, the retrospective design of the study inherently limits the generalizability of our findings. Secondly, the research was conducted at a single center with a cohort composed entirely of Caucasian patients, which may restrict the generalizability of the results to other populations or settings. Thirdly, the relatively small sample size of 67 patients could affect the robustness and statistical power of the study [the statistical power of the study, calculating by using G*Power software (version 3.1.9.6) [33], given a total sample size of 67 (with groups of 33 and 34 participants) a significance level of 0.05, and an effect size of 0.5, is approximately 0.65]. Additionally, the reliance on the RFT for assessing latent diplopia may not account for all potential functional deficits, suggesting the need for additional functional assessments in future research.

## 5. Conclusions

The present study identifies subclinical EM dysfunction and changes in inactive GO patients. Clinicians should consider comprehensive ocular evaluations, including imaging studies and functional tests like the RFT, which can be integrated into the standard VISA or EUGOGO evaluation to facilitate a better assessment of the extent of EOM involvement in GO. The early detection of latent diplopia and muscular abnormalities, as well as appropriate therapeutic options, might influence the progression and prognosis of more severe manifestations of late fibrotic alterations, thereby improving patient outcomes. Future research should focus on larger, multi-center studies with advanced imaging and functional assessments to gain a deeper understanding of EM dysfunction in inactive GO, and refine treatment approaches.

## Figures and Tables

**Table 1:** Demographic, laboratory and clinical data of patients.

	*All Patients (n = 67)* *n (%)*
***Gender***	
*Male*	21 (31.3%)
*Female*	46 (68.7%)
***Mean age (SD)***	46 (12.1)
***Thyroid status***	
*Hyperthyroid*	36 (53.7%)
*Euthyroid*	28 (41.8%)
*Hypothyroid*	3 (4.4%)
***Mean duration of disease (months)***	29.0 (41.21)
***Mean thyroid hormones (SD)***	
*TSH (mU/L)*	1.57 (3.02)
*FT4 (ng/dL)*	2.45 (3.90)
*FT3 (pg/mL)*	4.47 (4.47)
*TRAb title*	
*Negative*	16 (22.8%)
*Low* *(1–2 IU/L)*	22 (32.8%)
*Moderate (2–10 IU/L)*	16 (22.8%)
*High (>10 IU/L)*	13 (19.4%)
***ATA levels***	
*Positive (* *Anti-* *TG ≥ 4.5 IU/mL* *or* *Anti-TPO ≥ 60 IU/mL)*	48 (71.6%)
*Negative (* *Anti-* *TG < 4.5 IU/mL* *or* *Anti-TPO < 60 IU/mL)*	19 (28.4%)
***CAS***	
*CAS 0*	36 (53.7%)
*CAS 1*	26 (38.8%)
*CAS 2*	5 (7.4%)
***Exophthalmos (>21 mm or difference between the two eyes ≥ 3 mm)***	8 (11.9%)
***BCVA (logMAR) (SD)***	0.02 (0.09)
***Mean IOP (mmHg) (SD)***	
*Primary gaze*	14.46 (2.76)
*Up-gaze*	16.64 (4.07)
***US Medial Rectus***	
*Normal (<4 mm)*	69 (51.5%)
*Thickened (4–6 mm)*	57 (42.5%)
*Very Thickened (>6 mm)*	8(6.0%)
***US Lateral Rectus***	
*Normal (<4 mm)*	109 (81.3%)
*Thickened (4–6 mm)*	22 (16.4%)
*Very Thickened (>6 mm)*	3 (2.2%)

TSH = thyroid-stimulating hormone; FT4 = free thyroxine; FT3 = free triiodothy-ronine; TRAb = thyrotropin receptor antibody; Anti-TG = Anti-thyroglobulin; Anti-TPO = Anti-thyroid peroxidase; CAS = Clinical Activity Score; BCVA = Best-corrected visual acuity; IOP = Intraocular pressure; US = Ultrasound.

**Table 2:** Demographic, laboratory and clinical characteristics of two groups.

	*RFT-Positive (n = 33) n (%)*	*RFT-Negative (n = 34)* *n (%)*	*p-Value*
***Gender***			1 *
*Male*	10 (30.3%)	11 (32.4%)	
*Female*	23 (69.7%)	23 (67.6%)	
***Mean age (SD)***	49.1 (12.3)	43.0 (11.3)	0.03 ^†^
***Thyroid status***			0.75 ^χ^
*Hyperthyroid*	17 (51.5%)	19 (55.9%)	
*Euthyroid*	15 (45.5%)	13 (38.2%)	
*Hypothyroid*	1 (3.0%)	2 (5.9%)	
***Mean duration of disease (months) (SD)***	34.1 (37.6)	24.1 (44.5)	0.04 ^‡^
***Mean thyroid hormones (SD)***			
*TSH (mU/L)*	1.03 (1.27)	2.10 (4.08)	0.71 ^‡^
*FT4 (ng/dL)*	2.38 (3.17)	2.52 (4.54)	0.23 ^‡^
*FT3 (pg/mL)*	5.13 (5.85)	3.83 (2.45)	0.23 ^‡^
***TRAb title***			0.55 ^χ^
*Negative*	6 (18.2%)	10 (29.4%)	
*Low* *(1–2 IU/L)*	10 (30.3%)	12 (35.3%)	
*Moderate (2–10 IU/L)*	9 (27.3%)	7 (20.6%)	
*High (>10 IU/L)*	8 (24.2%)	5 (14.7%)	
***ATA levels***			1 *
*Positive (* *Anti-* *TG ≥ 4.5 IU/mL* *or* *Anti-TPO ≥ 60 IU/mL)*	24 (72.7%)	24 (70.6%)	
*Negative (* *Anti-* *TG < 4.5 IU/mL* *or* *Anti-TPO < 60 IU/mL)*	9 (27.3%)	10 (29.4%)	
***CAS***			0.30 ^χ^
*CAS 0*	18 (54.6%)	18 (53.0%)	
*CAS 1*	11 (33.3%)	15 (44.1%)	
*CAS 2*	4 (12.1%)	1 (2.9%)	
***Exophthalmos (>21 mm or difference between the two eyes ≥ 3 mm)***	3 (9.1%)	5 (14.7%)	0.71 *
***BCVA (logMAR)(SD)***	0.01 (0.01)	0.03 (0.01)	0.27 ^‡^
***Mean IOP (mmHg) (SD)***			
*Primary gaze*	14.20 (2.80)	14.71 (2.72)	0.46 ^‡^
*Up-gaze*	18.15 (4.56)	15.18 (2.87)	0.00005 ^‡^
***US Medial Rectus***			0.003 ^χ^
*Normal (<4 mm)*	27 (40.9%)	42 (61.8%)	
*Thickened (4–6 mm)*	31 (47.0%)	26 (38.2%)	
*Very Thickened (>6 mm)*	8 (12.1%)	0 (0.0%)	
***US Lateral Rectus***			0.10 ^χ^
*Normal (<4 mm)*	50 (75.8%)	59 (86.8%)	
*Thickened (4–6 mm)*	13 (19.7%)	9 (13.2%)	
*Very Thickened (>6 mm)*	3 (4.5%)	0 (0.0%)	

* = Fisher’s exact test; ^†^ = *t*-test; ^‡^ = Mann–Whitney U test; ^χ^ = Chi-squared test. TSH = thyroid-stimulating hormone; FT4 = free thyroxine; FT3 = free triiodothy-ronine; TRAb = thyrotropin receptor antibody; Anti-TG = Anti-thyroglobulin; Anti-TPO = Anti-thyroid peroxidase; CAS = Clinical Activity Score; BCVA = Best-corrected visual acuity; IOP = Intraocular pressure; US = Ultrasound.

## Data Availability

The data presented in this study are available on request from the corresponding author.

## References

[1] Brent G.A. (2008). Clinical practice. Graves’ disease. N. Engl. J. Med..

[2] Cooper D.S. (2003). Hyperthyroidism. Lancet.

[3] Weetman A.P. (2000). Graves’ Disease. N. Engl. J. Med..

[4] Eckstein A.K., Johnson K.T.M., Thanos M., Esser J., Ludgate M. (2009). Current insights into the pathogenesis of Graves’ orbitopathy. Horm. Metab. Res..

[5] Bartalena L., Tanda M.L. (2022). Current concepts regarding Graves’ orbitopathy. J. Intern. Med..

[6] Bartalena L., Tanda M.L. (2009). Clinical practice. Graves’ ophthalmopathy. N. Engl. J. Med..

[7] Scarabosio A., Surico P.L., Singh R.B., Tereshenko V., Musa M., D’Esposito F., Russo A., Longo A., Gagliano C., Agosti E. (2024). Thyroid Eye Disease: Advancements in Orbital and Ocular Pathology Management. J. Pers. Med..

[8] Bahn R.S. (2010). Graves’ Ophthalmopathy. N. Engl. J. Med..

[9] Smith T.J. (2010). Pathogenesis of Graves’ orbitopathy: A 2010 update. J. Endocrinol. Investig..

[10] Mourits M.P., Koornneef L., Wiersinga W.M., Prummel M.F., Berghout A., van der Gaag R. (1989). Clinical criteria for the assessment of disease activity in Graves’ ophthalmopathy: A novel approach. Br. J. Ophthalmol..

[11] Lennerstrand G., Tian S., Isberg B., Landau Högbeck I., Bolzani R., Tallstedt L., Schworm H. (2007). Magnetic resonance imaging and ultrasound measurements of extraocular muscles in thyroid-associated ophthalmopathy at different stages of the disease. Acta Ophthalmol. Scand..

[12] Nagy E.V., Toth J., Kaldi I., Damjanovich J., Mezosi E., Lenkey A., Toth L., Szabo J., Karanyi Z., Leovey A. (2000). Graves’ ophthalmopathy: Eye muscle involvement in patients with diplopia. Eur. J. Endocrinol..

[13] Jankauskiene J., Imbrasiene D. (2006). Investigations of ocular changes, extraocular muscle thickness, and eye movements in Graves’ ophthalmopathy. Medicina.

[14] Johnson B.T., Jameyfield E., Aakalu V.K. (2021). Optic neuropathy and diplopia from thyroid eye disease: Update on pathophysiology and treatment. Curr. Opin. Neurol..

[15] Prummel M.F., Bakker A., Wiersinga W.M., Baldeschi L., Mourits M.P., Kendall-Taylor P., Perros P., Neoh C., Dickinson A.J., Lazarus J.H. (2003). Multi-center study on the characteristics and treatment strategies of patients with Graves’ orbitopathy: The first European Group on Graves’ Orbitopathy experience. Eur. J. Endocrinol..

[16] Ponto K.A., Merkesdal S., Hommel G., Pitz S., Pfeiffer N., Kahaly G.J. (2013). Public health relevance of Graves’ orbitopathy. J. Clin. Endocrinol. Metab..

[17] Gräf M., Lorenz B. (2012). How to deal with diplopia. Rev. Neurol..

[18] Barrio-Barrio J., Sabater A.L., Bonet-Farriol E., Velázquez-Villoria Á., Galofré J.C. (2015). Graves’ Ophthalmopathy: VISA versus EUGOGO Classification, Assessment, and Management. J. Ophthalmol..

[19] Yoo H.S., Park E., Rhiu S., Chang H.J., Kim K., Yoo J., Heo J.H., Nam H.S. (2017). A computerized red glass test for quantifying diplopia. BMC Ophthalmol..

[20] Bitner D.P., Adesina O.O., Ding K., Farris B.K., Michael Siatkowski R. (2017). Comparison of Objective and Subjective Techniques of Strabismus Measurement in Adults With Normal Retinal Correspondence. J. Pediatr. Ophthalmol. Strabismus..

[21] Li X., Bai X., Liu Z., Cheng M., Li J., Tan N., Yuan H. (2021). The Effect of Inferior Rectus Muscle Thickening on Intraocular Pressure in Thyroid-Associated Ophthalmopathy. J. Ophthalmol..

[22] Karhanova M., Kovar R., Frysak Z., Sin M., Zapletalova J., Rehak J., Herman M. (2015). Correlation between magnetic resonance imaging and ultrasound measurements of eye muscle thickness in thyroid-associated orbitopathy. Biomed. Pap. Med. Fac. Univ. Palacky. Olomouc Czech Repub..

[23] Fledelius H.C., Zimmermann-Belsing T., Feldt-Rasmussen U. (2003). Ultrasonically measured horizontal eye muscle thickness in thyroid associated orbitopathy: Cross-sectional and longitudinal aspects in a Danish series. Acta Ophthalmol. Scand..

[24] Szelog J., Swanson H., Sniegowski M.C., Lyon D.B. (2022). Thyroid Eye Disease. Mo. Med..

[25] Yang J., Chen J., Shi B., You Y., Pi X., Zhao G., Jiang F. (2023). Effects of various extraocular muscle enlargement patterns on muscle diameter index in graves ophthalmopathy patients: A retrospective cohort study. Sci. Rep..

[26] Bharadwaj S.R., Hoenig M.P., Sivaramakrishnan V.C., Karthikeyan B., Simonian D., Mau K., Rastani S., Schor C.M. (2007). Variation of binocular-vertical fusion amplitude with convergence. Investig. Ophthalmol. Vis. Sci..

[27] Perros P., Crombie A.L., Matthews J.N., Kendall-Taylor P. (1993). Age and gender influence the severity of thyroid-associated ophthalmopathy: A study of 101 patients attending a combined thyroid-eye clinic. Clin. Endocrinol..

[28] Hesarghatta Shyamasunder A., Abraham P. (2017). Measuring TSH receptor antibody to influence treatment choices in Graves’ disease. Clin. Endocrinol..

[29] Nicolì F., Lanzolla G., Mantuano M., Ionni I., Mazzi B., Leo M., Sframeli A., Posarelli C., Maglionico M.N., Figus M. (2021). Correlation between serum anti-TSH receptor autoantibodies (TRAbs) and the clinical feature of Graves’ orbitopathy. J. Endocrinol. Investig..

[30] Tabasum A., Khan I., Taylor P., Das G., Okosieme O.E. (2016). Thyroid antibody-negative euthyroid Graves’ ophthalmopathy. Endocrinol. Diabetes Metab. Case Rep..

[31] Bartalena L., Kahaly G.J., Baldeschi L., Dayan C.M., Eckstein A., Marcocci C., Marinò M., Vaidya B., Wiersinga W.M., EUGOGO (2021). The 2021 European Group on Graves’ orbitopathy (EUGOGO) clinical practice guidelines for the medical management of Graves’ orbitopathy. Eur. J. Endocrinol..

[32] Lanzolla G., Marinò M., Marcocci C. (2021). Selenium in the Treatment of Graves’ Hyperthyroidism and Eye Disease. Front. Endocrinol..

[33] Kang H. (2021). Sample size determination and power analysis using the G*Power software. J. Educ. Eval. Health Prof..

